# Cost-effectiveness of ranibizumab for neovascular age-related macular degeneration

**DOI:** 10.1186/1478-7547-6-12

**Published:** 2008-06-24

**Authors:** Susan F Hurley, Jane P Matthews, Robyn H Guymer

**Affiliations:** 1 Bainbridge Consultants, 222/299 Queen St, Melbourne, VIC 3000, Australia; 2 School of Medicine, Griffith University, Australia; 3 School of Population Health, The University of Melbourne, Australia; 4 Macular Research Unit, Department of Ophthalmology, Centre for Eye Research Australia, The University of Melbourne, Australia

## Abstract

**Background:**

Intravitreal ranibizumab prevents vision loss and improves visual acuity in patients with neovascular age-related macular degeneration, but it is expensive, and efficacy beyond 2 years is uncertain.

**Methods:**

We assessed the cost-effectiveness of ranibizumab compared with no ranibizumab over 10 years, using randomized trial efficacy data for the first 2 years, post-trial efficacy assumptions, and ranibizumab acquisition costs ranging from the wholesale price ($1,950 per dose) to the price of bevazicumab ($50), a similar molecule which may be equally efficacious. We used a computer simulation model to estimate the probability of blindness, the number of quality-adjusted life-years (QALYs), direct costs (in 2004 U.S. dollars), and cost-effectiveness ratios for a 67-year old woman. Costs and QALYs were discounted at 3% per year.

**Results:**

The probability of blindness over 10 years was reduced from 56% to 34% if ranibizumab was efficacious for only 2 years, 27% if efficacy was maintained for a further 2 years only (base-case scenario), and 17% if visual acuity at 4 years was then sustained. It was cost-saving under all price assumptions, when caregiver costs were included. When caregiver costs were excluded, the cost per QALY for the base-case ranged from $5,600, assuming the bevazicumab price, to $91,900 assuming the wholesale ranibizumab price. The cost per QALY was < $50,000 when the cost of ranibizumab was less than $1000.

**Conclusion:**

From a societal perspective, ranibizumab was cost-saving. From a health care funder's perspective, ranibizumab was an efficient treatment when it cost less than $1000 per dose.

## Background

Ranibizumab's efficacy has been described as miraculous [1]. This humanized, recombinant, monoclonal antibody fragment is the first treatment for neovascular age-related macular degeneration that improves visual acuity. In 2 recent randomized controlled trials, MARINA (the Minimally Classic/Occult Trial of the Anti-VEGF Antibody Ranibizumab in the Treatment of Neovascular Age-Related Macular Degeneration)[2] and the ANCHOR study (Anti-VEGF Antibody for the Treatment of Predominantly Classic Choroidal Neovascularization in Age-Related Macular Degeneration) [3], it prevented vision loss and improved visual acuity.

The availability of ranibizumab is therefore likely to transform the management of neovascular macular degeneration, a disease that can be blinding and is epidemic in the developing world [1]. However, ranibizumab is expensive [4], its monthly intravitreal dosing regimen is inconvenient and potentially increases the risk of bacterial endophthalmitis [5], and its efficacy beyond 2 years is unknown. Before ranibizumab was licensed in the United States, some physicians treated patients with bevacizumab, a similar, but much cheaper, molecule which is licensed for the treatment of metastatic cancer of the colon or rectum [4]. Preliminary studies of bevacizumab's efficacy in neovascular macular degeneration suggest benefits similar to those of ranibizumab [4], so its "off label" use might continue, or it might be studied in randomized controlled clinical trials of patients with macular degeneration (and eventually licensed for this indication if found to be effective), or the price of ranibizumab might be reduced. There is also evidence that fewer than 24 monthly injections of ranibizumab may be just as efficacious, and trials are underway evaluating less frequent dosing, and variable dosing regimens guided by visual acuity and optical coherence tomography findings [1,5,6].

The purpose of the present analysis was to perform incremental cost-effectiveness analyses of the use of ranibizumab for neovascular macular degeneration. These analyses investigated ranibizumab's efficiency in terms of improved vision and quality of life outcomes, compared with current standard management, and the extent to which the initial cost of ranibizumab will be offset by savings due to prevention of vision loss.

## Methods

### Model Overview

We developed a Markov model using the decision analysis software TreeAge[7] to simulate the progression of neovascular age-related macular degeneration in patients in the United States and to predict the following outcomes associated with a ranibizumab treatment strategy and a no ranibizumab treatment strategy (i.e. standard or usual care): the probability of blindness, number of blind-years (years spent blind), number of quality-adjusted life-years (QALYs), and direct costs (excluding patient time and travel costs) from a societal perspective and from a health care funder's perspective in 2004 U.S. dollars. We compared the 2 strategies in terms of incremental cost-effectiveness ratios over time horizons of up to 10 years. Note that in the United Kingdom, the incremental cost per QALY would sometimes be referred to as a cost-utility ratio. In this paper, we refer to cost per QALY, cost per case of blindness averted and cost per blind-year averted as cost-effectiveness ratios, an approach that is standard in the United States.

**Figure 1:**
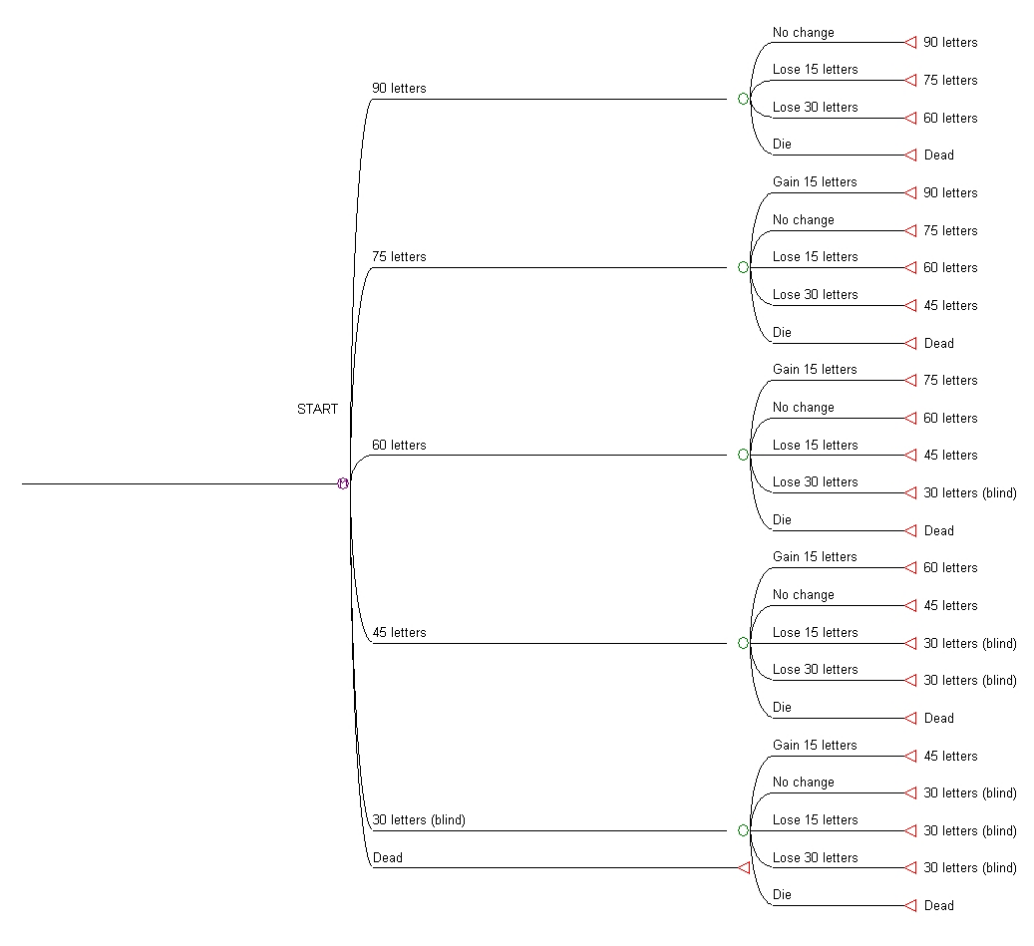
Simplified diagram of Markov tree model, which was analysed for the Ranibizumab treatment strategy and the No ranibizumab treatment strategy.

Disease progression was characterized by a series of annual transitions between health states, defined by the patient's visual acuity and measured in terms of the number of letters read by the better seeing eye on the logMAR chart [8]. Our model was therefore a "second eye" model, i.e. the other ("first") eye was assumed to have worse vision, and therefore after treatment with ranibizumab QALYs accrued immediately. The 5 health states considered were referred to as 90, 75, 60, 45 and 30 letters, and corresponded to the number of letters read being > 85, 70–80, 55–65, 40–50, and < 35, respectively. We assumed that, each year, a patient's visual acuity would either increase by 15 letters, remain the same, decrease by 15 letters, or decrease by 30 letters. (See Figure 1 for a simplified version of the model). The probabilities of these events did not depend on the number of letters able to be read at the start of the year. A patient was classified as blind when they moved to the 30 letters state, corresponding to a visual acuity of < 35 letters read (Snellen equivalent < 20/200). In the United States, and many other countries, legal blindness is defined as visual acuity of ≤ 20/200 in the better eye with the best correction [9,10].

Our assumptions about ranibizumab's efficacy, and hence the annual transition probabilities between health states, were based on the results of MARINA[2], rather than the ANCHOR trial [3], because MARINA outcomes were reported at 2 years follow-up [2], compared to only 12 months for the ANCHOR trial [3], and, at 12 months follow-up, the efficacy of ranibizumab compared to sham injection in MARINA was virtually identical to the efficacy of ranibizumab compared with verteporfin photodynamic therapy assessed in the ANCHOR trial [5]. Furthermore, approximately three-quarters of patients with neovascular macular degeneration have the type of disease studied in MARINA (minimally classic or occult choroidal neovascularization) [2,11], compared with one-quarter of patients who have predominantly classic disease [11], which was studied in the ANCHOR trial [3]. In MARINA, other accepted therapies were permitted, including verteporfin photodynamic therapy in line with reimbursement guidelines, so our analysis compared ranibizmab treatment with standard care without ranibizumab.

Beyond the 2-year time horizon of MARINA we considered 3 hypothetical scenarios underpinned by different assumptions about disease progression, and ranibizumab's effectiveness and dosing regimen (Table 1). We assumed that transition probabilities between visual acuity health states for the no ranibizumab strategy would be the same for all 3 scenarios. In the "base-case" ranibizumab scenario we assumed that the changes in visual acuity associated with ranibizumab in the second year of MARINA would continue for the next 2 years, and that patients would then develop the atrophy form of macular degeneration [12]. We assumed that ranibizumab would be given according to the MARINA dosing regimen[2] for the first 2 years, then every 3 months for Years 3 and 4, then ceased. For the "sustained-effect" scenario, we again assumed that the effect of ranibizumab observed in the second year of MARINA would continue for the next 2 years, but that visual acuity at the end of the 4-year time horizon would be sustained. We assumed that ranibizumab would be given less frequently than in MARINA and for only 2 years. For the "non-sustained-effect" scenario, we assumed that ranibizumab would be given according to the MARINA dosing regimen for 2 years, but that it would be ineffective beyond that time and would therefore be ceased and visual acuity would decline at the same rate as in the no ranibizumab treatment strategy.

**Table 1:** Assumptions for annual transition probabilities, and ranibizumab dosing regimen

	**Ranibizumab treatment**			**No ranibizumab treatment**
	**Base-case scenario**	**Sustained-effect scenario**	**Non-sustained-effect scenario**	
Annual transition probabilities*				
Time horizon				
Years 1 and 2	Results of MARINA, 0.5 mg ranibizumab arm.	As for base-case	As for base-case	Results of MARINA, sham arm.
Years 3 and 4	Year 2 MARINA data, 0.5 mg ranibizumab arm.	As for base-case	Year 2 MARINA data, sham arm	Year 2 MARINA data, sham arm.
Years 5 to 10	Year 5 to 10 progression rates of the geographic atrophy form of age-related macular degeneration	No further transitions (neither increasing nor decreasing visual acuity)	Year 2 MARINA data, sham arm, progression rates decreasing by 40% each year	Year 2 MARINA data, sham arm, progression rates decreasing by 40% each year
Ranibizumab dosing regimen				
	One dose monthly for the first 2 years, then every 3 months until end of Year 4. No ranibizumab thereafter.	Three doses at monthly intervals, then every 3 months until the end of Year 2. No ranibizumab thereafter.	One dose monthly for the first 2 years. No ranibizumab thereafter.	-

MARINA: Minimally Classic/Occult Trial of the Anti-VEGF Antibody Ranibizumab in the Treatment of Neovascular Age-Related Macular Degeneration[2]

The model also incorporated all causes mortality, the probability of which did not vary with visual acuity.

### Estimates for Model Variables

#### Visual acuity before treatment

The distribution of visual acuity for patients with neovascular age-related macular degeneration at the time of a decision to treat or not treat with ranibizumab was based on the distribution of visual acuity of patients randomized to treatment in the Minimally Classic/Occult Trial of the Anti-VEGF Antibody Ranibizumab in the Treatment of Neovascular Age-Related Macular degeneration (MARINA), as detailed in Table 2[2].

**Table 2:** Estimation of initial distribution of visual acuity for the model from MARINA data [2].

**MARINA**		**Model**	
Visual acuity*	**Number (%) of patients^†^**	**Visual acuity (letters)**	**Proportion of patients**

		90	0%
20/40 or better (≥ 70 letters)	99 (13.8%)	75	14%
Better than 20/200 but worse than 20/40 (>35 but < 70 letters)*	519 (72.5%)^‡^	60	36%
		45	36%
20/200 or worse (≤ 35 letters)	98(13.7%)	30	14%

* The eligibility criteria for the trial required patients to have a best corrected visual acuity of between 20/40 (70 letters) and 20/320 (25 letters).

^† ^Data from the three treatment arms were pooled.

‡ Half the patients in this visual acuity group were assigned to the 60 letter state in the Model and half were assigned to the 45 letter state.

#### Transition probabilities between health states

The annual probabilities of death were the estimated age-group and sex-specific all causes mortality rates for the United States for 2004 [13]. The annual transition probabilities between the other health states were estimated from the MARINA trial data [2]. Data for patients randomized to receive 0.5 mg of ranibizumab monthly (the dose subsequently licensed by the Food and Drug Administration) were used for the ranibizumab treatment strategy, and data for patients who received the sham injection were used for the no ranibizumab strategy.

Rosenfeld et al.[2] reported the probabilities of an increase in visual acuity by ≥ 15 letters, a loss of < 15 letters and a loss of ≥ 30 letters from baseline at 12 and 24 months. We took the probability of a 15 letter gain in visual acuity to be the probability of an increase in visual acuity by ≥ 15 letters, and the probability of a loss of 30 letters to be the probability of a loss of ≥ 30 letters. We calculated the probability of no change in visual acuity by subtracting the probability of an increase of ≥ 15 letters from the probability of a loss of < 15 letters, and the probability of a loss of 15 letters by subtracting the sum of the probabilities of a loss of < 15 letters and a loss of ≥ 30 letters from 1.

The annual transition probabilities for the first year were simply the cumulative probabilities at the end of 12 months. We estimated transition probabilities for the second year by expressing the cumulative probabilities at 24 months in terms of the cumulative probabilities at 12 months and the probabilities of gaining 15 letters or of losing 0, 15 or 30 letters in the second year and solving the resulting equations. The transition probabilities were assumed to be independent of the number of letters read at the beginning of the year.

All estimated probabilities had values between 0 and 1, except for the estimated value of the probability of gaining 15 letters for the Sham arm which was -0.008. This value was set to 0 and the value of the probability of losing 0 letters was decreased by 0.008 for consistency. With these revised estimates (shown in Table 3), the calculated values of the cumulative probabilities at the end of the second year differed from the reported values by less than 0.5%.

**Table 3:** Annual transition probabilities for ranibizumab and no ranibizumab treatment strategies for first two years

	**Difference in number of letters read on logMAR chart at beginning and end of year**							
	
	**Ranibizumab treatment**				**No ranibizumab treatment**			
**Year**	**15**	**0**	**-15**	**-30**	**15**	**0**	**-15**	**-30**

1	0.338	0.608	0.042	0.012	0.050	0.572	0.235	0.143
2	0.030	0.902	0.051	0.017	0.000	0.848	0.070	0.082

Annual transition probabilities between visual acuity health states for the geographic atrophy form of age-related macular degeneration were needed for the "base-case" scenario (see Table 1). We assumed that each year following a diagnosis of geographic atrophy there would either be no change in visual acuity, a loss of 15 letters or a loss of 30 letters, and that the probabilities of these 3 events did not depend on the number of letters able to be read at the start of the year. For the first 4 years following diagnosis these annual transition probabilities were estimated from the natural history data reported by Sunness et al.[14]

**Figure 2:**
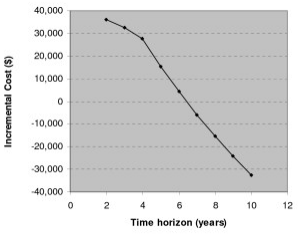
Incremental costs versus time for ranibizumab treatment compared with no ranibizumab treatment, assuming the base-case scenario and the wholesale price for ranibizumab, and including caregiver costs.

We combined the data from visual acuity (VA) group 1 (67 cases with VA > 20/50) and group 2 (43 cases with VA ≤ 20/50 and > 20/200). Because of the small number of cases available, we assumed that the transition probabilities would be the same in each of the first 4 years. The cumulative percentages of eyes with visual acuity worsening by 3 or more lines (15 or more letters) after 4 years of follow-up were obtained from Figure 2 (70% for VA group 1 and 43% for VA group 2). A weighted average of these cumulative percentages was obtained with weights proportional to the number of cases in each group, leading to the combined estimate of 59.4%. The cumulative percentages of eyes with visual acuity worsening by 6 or more lines (30 or more letters) after 4 years of follow-up were obtained from the text (45% for VA group 1 and 20% for VA group 2). Again, a weighted average of this cumulative percentage was obtained, leading to the combined estimate of 34.8%. The cumulative percentage of eyes with worsening visual acuity (VA) by 3 or more lines but not by 6 or more lines was obtained by subtraction (59.4% – 34.8% = 24.6%). The annual transition probabilities were then obtained by expressing the cumulative probabilities of losing 15 or 30 letters from baseline after 4 years of follow-up in terms of these annual transition probabilities and solving the resulting equations. Thus, the estimated annual probability of losing 15 letters was 0.121 and the estimated annual probability of losing 30 letters was 0.081.

For the subsequent years (year 5 and onwards) we assumed that the annual transition probabilities of losing 15 or 30 letters would be reduced by 40% each year, i.e., the year 5 probabilities would be 60% of the year 4 probabilities, and the year 6 probabilities would be 60% of the year 5 probabilities, and so on.

#### Costs and utilities

We used 2 estimates for the cost of a 0.5 mg dose of ranibizumab: $1,950, the wholesale acquisition price, and $50, the maximum cost of a molar equivalent dose of bevacizumab, dispensed into a syringe for intraocular use [4]. An administration cost of $250 was added to both estimates [4].

The other cost estimates and utility values for the Model are summarized in Table 4[15-18]. We considered 3 categories of cost apart from ranibizumab: the cost of medical care for age-related macular degeneration, the cost of medical care attributable to vision loss, and the cost of caregiving by family, friends and professional carers. These costs did not vary with the treatment strategy. The macular degeneration medical care costs were sourced from an analysis of Medicare files [15]. The annual cost per patient in 2001 for 6,558 beneficiaries with wet (neovascular) disease who did not receive photodynamic therapy was used. Vision loss is associated with an increased risk of falls [19]; vehicular accidents, depression and nursing home placement [16]; and longer hospital stays [20]. We used estimates of the excess average annual medical cost between 1999 and 2003 for non-eye related medical care attributable to vision loss for over 24,000 Medicare beneficiaries (Table 4) [16]. Both categories of medical care cost were converted to year-2004 U.S. dollars on the basis of the medical care component of the Consumer Price Index [21]. Estimates of the cost of caregiving were sourced from a survey of 803 patients which found that use of paid and unpaid help increased significantly as visual acuity declined, and that around 72% of assistance was provided by the patient's spouse or family members [17]. The hourly cost of care was valued at the June 2004 non-farm, non-supervisory seasonally adjusted wage from the Bureau of Labour and Statistics, irrespective of whether caregivers were paid or unpaid. Utility estimates were based on those obtained using the time-trade off method in a study of 81 patients with macular degeneration and vision loss [18]. The transfer cost of disability payments for patients who become blind were not considered.

**Table 4:** Estimates for cost and utility model variables

	**Health states (Visual acuity measured in letters read)**				
	**90**	**75**	**60**	**45**	**≤ 30**
Annual costs*					
Medical care					
AMD -related^†^	$645	$645	$645	$645	$645
Non eye-related^‡^	-	-	$2,288	$2,288	$3,445
Total medical care	$645	$645	$2,933	$2,933	$4,090
Caregivers^§^	-	$795	$3,625	$14,185	$47,086
Total	$645	$1,440	$6,558	$17,118	$51,176
Utility values^¶^					
	0.89	0.89	0.81	0.57	0.52

* 2004 U.S. dollars

† AMD = age-related macular degeneration. Excluding costs of ranibizumab acquisition and administration. Source: Halpern et al.[15]

‡ Source: Javitt et al.[16]

§Source: Schmier et al.[17]

¶Source: Brown et al.[18]

### Analysis

Future costs, blind-years and QALYs were discounted at 3% per year [22]. The only model parameter that varied with gender and age was all-causes mortality. We analysed the model for 67 and 77-year old women and men, the mid-point of the relevant 5-year age groups, and detailed results are presented for a 67-year old woman. Age-related macular degeneration occurs in people aged 65 years and over, and a new, effective treatment such as ranibizumab is likely to be prescribed soon after symptoms lead to diagnosis.

We first assessed effectiveness and cost-effectiveness over the 2-year time horizon of MARINA. The 3 scenarios underpinned by different assumptions about ranibizumab's subsequent effectiveness and dosing regimen were then analysed over 10 years. Analyses were conducted for each ranibizumab acquisition cost assumption, both including and excluding caregiver costs. For the base-case scenario, sensitivity analyses were conducted to assess, first, the impact of time horizons between 2 and 10 years on the incremental cost of ranibizumab treatment including the cost of caregiving, and, second, the impact on the incremental cost per QALY, excluding caregiver costs, of varying the ranibizumab acquisition cost between the 2 extremes.

Costs, QALYs and blind-years were estimated directly from Markov cohort analyses. The probability of becoming blind was estimated using Monte Carlo simulation, in which the clinical course of 10,000 patients was simulated, one at a time, over the specified time horizon.

## Results

Over a 2-year period, based on MARINA results, the probability of becoming blind for a 67-year old woman was 39% without ranibizumab treatment, and was reduced to 14% with ranibizumab. Ranibizumab treatment was associated with a decrease of 0.31 in the expected number of blind-years and an increase of 0.118 QALYs. The QALY gain was slightly lower for 67-year old men (0.116 QALYs) and 77-year old women (0.114 QALYs) because of their lower life expectancy.

The costs and cost-effectiveness of ranibizumab treatment based on these health outcomes at 2-years are summarised in Table 5. When caregiver costs were included and the ranibizumab acquisition cost was taken to be the bevacizumab price, ranibizumab treatment cost less than the no ranibizumab strategy (i.e. it was dominant). When the ranibizumab cost was assumed to be its wholesale price, the ranibizumab strategy cost about $36,300 more than the no ranibizumab strategy, and the incremental cost per QALY gained was over $300,000. When caregiver costs were excluded, the incremental cost per QALY associated with ranibizumab treatment was $50,400 at the bevacizumab price, and over $400,000 at the ranibizumab wholesale price. The cost-effectiveness profile was similar for 67-year old men and 77-year olds, but cost-effectiveness ratios were slightly higher because of reduced life expectancy.

**Table 5:** Costs, incremental costs and cost-effectiveness ratios* for ranibizumab treatment compared with no ranibizumab treatment for a 67-year old woman over the 2-year time horizon of MARINA†

	**Cost**			**Cost per case of blindness prevented**	**Cost per blind-year prevented**	**Cost per QALY gained**
	**Ranibizumab treatment**	**No ranibizumab treatment**	**Difference**			
	**$**	**$**	**$**	**$**	**$**	**$**
Including caregiver costs						
Ranibizumab cost						
Wholesale price	78,900	42,700	36,300	145,400	116,500	308,400
Bevacizumab price	34,000	42,700	-8,700	Dominant^‡^	Dominant	Dominant
Excluding caregiver costs						
Ranibizumab cost						
Wholesale price	56,700	5,800	50,900	204,100	163,500	432,900
Bevacizumab price	11,700	5,800	6,000	23,800	19,000	50,400

*Costs are in 2004 U.S. dollars and were rounded. Costs, blind-years and QALYs were discounted at 3% per annum

† Rosenfeld et al.[2]

‡ Dominant: The ranibizumab treatment strategy was more effective and cost less than the no ranibizumab strategy

At 10 years, the cumulative probability of blindness was 56% for the no ranibizumab treatment strategy, and was reduced to 34%, 27%, or 17% with ranibizumab under the non-sustained-effect, base-case and sustained- effect scenarios, respectively. The expected number of blind-years was reduced from 3.74 to 1.61 under the base-case, and 1.27 and 2.03, respectively, under the sustained-effect and non-sustained-effect scenarios. The number of QALYs was increased from 4.9 to 5.69 under the sustained-effect scenario, 5.58 for the base-case and 5.45 for the non-sustained-effect scenario.

Table 6 summarizes the cost outcomes over 10-years. When caregiver costs were included, the ranibizumab treatment strategy was cost saving (dominant) under all assumptions. When caregiver costs were excluded, the ranibizumab strategy was also dominant under the sustained-effect scenario, assuming the bevacizumab price, and cost $20,300 per QALY and $6,400 per blind-year prevented assuming the ranibizumab wholesale price. Under the base-case scenario, the cost per case of blindness prevented varied from $13,200 to $217,700, and the cost per QALY gained varied from $5,600 to $91,900, depending on the ranibizumab cost. For the non-sustained-effect scenario, excluding caregiver costs, the cost of the ranibizumab strategy was lower than for the base-case, and the cost-effectiveness ratios were therefore slightly lower than those for the base-case. This was because the base-case assumed 2 more years of ranibizumab treatment than the non-sustained-effect scenario, and the medical care cost savings associated with the predicted additional reduction of 0.42 blind-years was not enough to offset the additional ranibizumab cost.

**Table 6:** Costs, incremental costs and cost-effectiveness ratios* for ranibizumab treatment compared with no ranibizumab treatment for a 67-year old woman over a 10-year time horizon, under different treatment effectiveness and dosing scenarios.

**Scenario**	**Cost**			**Cost per case of blindness prevented**	**Cost per blind-year prevented**	**Cost per QALY gained**
	**Ranibizumab treatment**	**No ranibizumab treatment**	**Difference**			
	**$**	**$**	**$**	**$**	**$**	**$**
**Base-case scenario**						
Including caregiver costs						
Ranibizumab cost						
Wholesale price	205,800	238,300	-32,500	Dominant^†^	Dominant	Dominant
Bevacizumab price	147,100	238,300	-91,100	Dominant	Dominant	Dominant
Excluding caregiver costs						
Ranibizumab cost						
Wholesale price	88,800	26,300	62,400	217,700	29,200	91,900
Bevacizumab price	30,100	26,300	3,800	13,200	1,800	5,600
**Sustained-effect scenario**						
Including caregiver costs						
Ranibizumab cost						
Wholesale price	144,400	238,300	-93,800	Dominant	Dominant	Dominant
Bevacizumab price	125,500	238,300	-112,700	Dominant	Dominant	Dominant
Excluding caregiver costs						
Ranibizumab cost						
Wholesale price	42,200	26,300	15,900	41,100	6,400	20,300
Bevacizumab price	23,300	26,300	-3,000	Dominant	Dominant	Dominant
**Non-sustained-effect scenario**						
Including caregiver costs						
Ranibizumab cost						
Wholesale price	209,800	238,300	-28,500	Dominant	Dominant	Dominant
Bevacizumab price	164,800	238,300	-73,500	Dominant	Dominant	Dominant
Excluding caregiver costs						
Ranibizumab cost						
Wholesale price	74,000	26,300	47,700	218,600	27,900	86,900
Bevacizumab price	29,100	26,300	2,700	12,500	1,600	5,000

*Costs are in 2004 U.S. dollars and were rounded.

Costs, blind-years and QALYs were discounted at 3% per annum

† Dominant: Ranibizumab treatment was more effective and cost less than the no ranibizumab strategy

**Figure 3:**
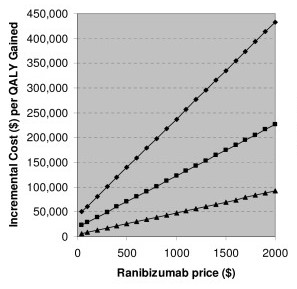
Incremental cost per QALY versus ranibizumab price for 3 time horizons, assuming the base-case scenario.

The sensitivity analysis on the analytic time horizon (Figure 2) showed that, when caregiver costs were included, the ranibizumab treatment strategy was cost-saving beyond 6 years, even at the wholesale price. The sensitivity analysis on ranibizumab cost (Figure 3) showed that ranibizumab treatment reached a threshold cost-effectiveness of $50,000 per QALY at about $1000 per dose over 10-years, $300 per dose over 4-years and just less than $50 over a 2-year time horizon.

## Discussion

From a societal perspective, the time spent by family, friends and professionals caring for people who are blind is relevant when evaluating a treatment that prevents vision loss. When the costs of such caregiving were included in this analysis, and a dose of ranibizumab was costed at the price of bevacizumab, the ranibizumab treatment strategy cost less than no treatment, even over a time horizon of 2 years. When the cost of ranibizumab was taken to be its current wholesale price, ranibizumab treatment was cost-saving over time horizons longer than 6 years.

Many health care funders will, however, make decisions about the provision of ranibizumab from the perspective of their own organisation, and will not consider the cost of non-medical care for people with poor visual acuity. When caregiver costs were excluded from our analyses, ranibizumab treatment was cost-saving at 10-years under only one set of assumptions: sustained-effectiveness, administration of ranibizumab less frequently for only 2 years, and the ranibizumab cost equal to the bevacizumab price (Table 6). However, treatments do not have to be cost-saving to be considered "cost-effective". Although there is no universally accepted threshold cost-effectiveness ratio below which an intervention is viewed as cost-effective, Britain's National Institute for Health and Clinical Excellence (NICE), for example, is increasingly likely to reject technologies on the basis of cost-ineffectiveness when their cost per QALY is between US$30,000 and US$45,900 (between approximately £20,000 and £30,000 per QALY) [23]. This suggests that NICE definitely regards interventions with cost-effectiveness ratios of less than $30,000 per QALY as cost-effective, and may regard those with ratios between $30,000 and around $50,000 per QALY as cost-effective. In contrast, Murray et al. recently defined "very cost-effective" interventions as those that gain each year of healthy life at a cost less than the gross domestic product (GDP) per head, and "cost-effective" interventions as those that gain a year of healthy life at a cost of between one and 3 times GDP per head [24]. Murray and colleagues' analysis was based on disability-adjusted life-years (DALYs), which are similar, but not identical, to QALYs [25]. Nevertheless, some inferences about the "cost-effectiveness" of ranibizumab can be made on the basis of Murray et al.'s analysis. The GDP per person in the U.S. was $43,500 in 2006 [26]. Ranibizumab could therefore be regarded as very cost-effective when its cost per QALY was less than about $50,000, and cost effective when this ratio was less than about $130,000. At the 10-year time horizon, ranibizumab could therefore be regarded as cost-effective according to Murray and colleagues' criteria, but would be unlikely to be recommended by NICE if the price per dose was greater than about $1,000.

Detsky and Laupacis recently drew attention to speculation that the identification of threshold cost-effectiveness ratios for funding could encourage drug companies to charge a price that achieves that ratio, even if the drug could be sold profitably at a lower price [27]. However, our results could in fact be used by funding agencies in the United States to determine the price at which ranibizumab falls below their own cost-effectiveness threshold for funding (see Figure 3), and to underpin price negotiations with the manufacturer. An alternative approach to implementing cost-effective treatment for neovascular age-related macular degeneration would be to conduct a randomized controlled trial of bevazicumab relative to ranibizumab. A UK-based economic analysis used modelling to determine the efficacy profile bevazicumab would need to have to be regarded as cost-effective [28]. This approach is likely to take far longer than price negotiations for ranibizumab.

NICE did in fact conduct an appraisal of ranibizumab for neovascular age-related macular degeneration, which included an independent economic evaluation [29]. This evaluation used the United Kingdom price of £761.20 for a ranibizumab injection, which, when converted to US dollars on the basis of the Gross Domestic Product Purchasing Power Parity [30], is about 60% of the US wholesale price. Other costs were derived largely from clinical guidelines and discussions with specialists. The evaluation's estimate for the annual cost of medical, community and residential care for a patient who becomes blind was only one-fifth of the cost estimate derived from US experience that we used in our study. NICE recommended ranibizumab for the treatment of all forms of neovascular age-related macular degeneration included on its marketing authorisation, under specified clinical conditions, and provided the cost of treatment beyond 14 injections is met by ranibizumab's manufacturer. The incremental cost-effectiveness ratio for ranibizumab treatment of minimally classic or occult no classic lesions, over a 10 year follow-up period and assuming only 14 injections are administered was £19,904 per QALY, which is consistent with the thresholds outlined above.

Our analysis had a US context. It was underpinned by the results of a high quality randomized trial [2], US cost data from the Medicare files [15,16], caregiver cost and utility data from US studies of patients with macular degeneration [17,18], and explicit assumptions about dosing regimens and post-trial efficacy. We did not include adverse events associated with ranibizumab in our model as none occurred with increased frequency in MARINA[2] or the ANCHOR study [3]. However, an analysis of combined data from the 2 trials suggested that ranibizumab increased the risk of nonocular hemorrhage [31], and this possibility is being monitored in a long-term extension study and ongoing trials [32]. All patients in both trials received intravitreal injections, but serious uveitis occurred in only 7 of the 1,139 patients (0.6%)[2,3] Including this very rare event in the model would not impact predicted health or cost outcomes. Similarly, any disutility associated with the intravitreal injections, for example due to pain, was not considered because the duration of the injections and any consequential decrease in the utility of life was too small to have any impact on the cost-effectiveness ratios.

Beyond 2 years, our model was based on 3 sets of assumptions about treatment effectiveness and dosing, as trial data were unavailable. In contrast, the independent economic evaluation commissioned by NICE [33], and the bevazicumab modelling study by Raftery and colleagues[28] assumed that after a two-year ranibizumab treatment period visual acuity would decline at the same rate as observed in the control group. Each of the three scenarios we modelled is likely to reflect the experience of at least some patients. Data from ongoing studies will clarify the average long-term outcomes of ranibizumab therapy.

## Conclusion

Notwithstanding the uncertainty about long-term outcomes, our study demonstrated that over a 10-year time horizon, under all plausible assumptions, ranibizumab was cost-saving from a societal perspective. From a health care funder's perspective, ranibizumab was cost-effective over a 10-year time horizon when it cost $1000 per dose or less (about half the current wholesale price). Ranibizumab would be cost-effective at a higher price in settings where cost-effectiveness ratios higher than $50,000 per QALY were regarded as acceptable.

## Competing interests

The authors declare that they have no competing interests. Associate Professor Guymer has been an investigator on clinical trials funded by Novartis Australia. This study was carried out completely independently of Novartis. We did not inform Novartis that it was being conducted and they did not provide any funding.

## Authors' contributions

All authors participated in designing the study. SFH sourced the data, JPM programmed and ran the Markov model, SFH drafted the manuscript, and all authors participated in critically revising the manuscript and approved the final version.
